# Direct Nuclear Delivery of Proteins on Living Plant via Partial Enzymatic Cell Wall Digestion

**DOI:** 10.3390/cimb46120870

**Published:** 2024-12-23

**Authors:** Qufei Gu, Nathan Ming, Yalikunjiang Aizezi, Xiaoyang Wei, Yizhong Yuan, Brian Esquivel, Zhi-Yong Wang

**Affiliations:** 1 Department of Plant Biology, Carnegie Institution for Science, Stanford, CA 94305, USA; nming@carnegiescience.edu (N.M.); yaizezi@carnegiescience.edu (Y.A.); xwei@carnegiescience.edu (X.W.); yyuan@carnegiescience.edu (Y.Y.); besquivel@carnegiescience.edu (B.E.); 2 Department of Physics, Stanford University, Stanford, CA 94305, USA; 3 Department of Biology, Stanford University, Stanford, CA 94305, USA

**Keywords:** green fluorescent protein, nuclear localization sequence, peptide, cell wall, partial enzymatic digestion

## Abstract

Nuclear protein delivery underlies an array of biotechnological and therapeutic applications. While many variations of protein delivery methods have been described, it can still be difficult or inefficient to introduce exogenous proteins into plants. A major barrier to progress is the cell wall which is primarily composed of polysaccharides and thus only permeable to small molecules. Here, we report a partial enzymatic cell wall digestion-mediated uptake method that efficiently delivers protein into the nucleus of plant cells. Such a method allowed efficient nuclear delivery of green fluorescent protein (GFP) flanked by two nuclear localization sequences (NLS) into *Arabidopsis thaliana* epidermal root cells without the usual need for large doses of nanoparticles or tissue cultures. We also show that switching from daylight to far-red light-grown conditions promotes effective protein penetration into deep cell layers. This study establishes that a partial enzymatic cell wall degradation could be applied to other cell organelles by changing the localization sequence, paving the way toward the rational engineering of plants.

## 1. Introduction

The delivery of exogenous biomolecules into walled plant cells enables targeted genetic engineering of plants. While several tools exist for the delivery of nucleic acids in plants, very few enable the delivery of proteins into walled plant cells. The development of CRISPR-Cas9 [1,2,3,4] and other emerging genome editing tools [5] has not only revolutionized the field of plant transformation, but also further increased the demands on working protein delivery tools. However, it is still difficult or inefficient to introduce proteins into plants, and as a result, many methods including biolistic bombardment, microinjection [6], and electroporation require tissue culture to generate stably transformed plants [7,8,9]. Tissue culture is time-consuming, laborious, cost-intensive, and cannot be successfully performed for all organisms or cultivars. Although synthetic carriers such as nanomaterials [10,11,12] and cell-penetrating peptides (CPPs) [11,13,14] allow proteins to cross the rigid and multilayered cell wall and phospholipid bilayer, the applications of these platforms in plant genetic engineering are limited due to low reproducibility and efficiency. A well-known challenge is that these carrier-based methods often have strong platform-to-platform, batch-to-batch, and even device-to-device variabilities, especially in light of the growing evidence that nanoparticles (NPs) are not required for biomolecule delivery in plants but instead provide protection against degradation [15]. Another major barrier to the use of nanotechnology is the lack of quantitative validation of successful intracellular protein delivery, which is difficult to distinguish from artifact and lytic sequestration [12]. New methods need to be invented to make protein delivery rapid and efficient for some plant species and possible for other species that cannot yet be accomplished using existing methods. Unlike mammalian cells, the plant cell cytosol is tightly compressed against the cell wall by the vacuole, which makes it difficult to perform unambiguous imaging of cytosolic contents. Moreover, plant cells are heterogeneous in shape and have many auto-fluorescent bodies, making distinguishing signals from background noise challenging. Here, we provide a novel approach for delivering proteins into plant cells by using partial enzyme digestion of plant cell walls. Our hypothesis is that the delivery of proteins into plant cells does not need to be preceded by the complete removal of the cell wall. By optimizing the various conditions, including enzyme types and growth conditions, we achieved direct nuclear protein delivery throughout all cell layers. As a proof of concept, we used GFP flanked by two NLS motifs (NLS-GFP-NLS-His) to assess the functionality of PECWD to detect successful nuclear GFP delivery. We utilized 3-day-old wild-type Arabidopsis seedlings due to their developing immature cell walls when compared to the more rigid structure of the mature cell walls of older seedlings or adult plants. Moreover, the miniature dimensions can accommodate confocal imaging and address the need for scaling up.

## 2. Materials and Methods

### 2.1. Materials

Hemicellulase and pectinase were purchased from Sigma-Aldrich Inc. (St. Louis, MO, USA). Macerozyme was obtained from GoldBio Tech. Co. (St. Louis, MO, USA). All gene fragments were synthesized by Twist Bioscience (San Francisco, CA, USA). Phusion High-Fidelity DNA Polymerase was purchased from Thermo Fisher Scientific Inc. (Waltham, MA, USA). Restriction enzymes and a Gibson assembly kit were obtained from New England Biolabs (Ipswich, MA, USA). *Escherichia coli* (*E. coli*) BL21 strain harboring pET28a-Cas9-His was purchased from Addgene Inc. (Watertown, MA, USA). Unless otherwise stated, all chemicals were obtained from Fisher Scientific Inc. (Pittsburgh, PA, Waltham, MA, USA).

### 2.2. Recombinant Protein Expression and Purification

In vitro expression of His-tagged GFP protein was described previously [16]. The GFP coding sequence flanked by two SV40 NLS motifs was inserted into pET28a (+) to generate pET28a-NLS-GFP-NLS-His using Gibson Assembly. The recombinant vector was transformed into an *E. coli* Top-10 strain for plasmid extraction and sequencing. The pET28a plasmid-carrying coding sequence was transformed into BL21(DE3) *E. coli* strains for protein expression. A single transformed clone was cultured in 10 mL LB medium overnight. The culture was transferred into 2L LB medium for large-scale induction. *E. coli* was grown to OD_600_ = 0.8 at 37 °C with shaking and cooled to 16 °C before induction. Protein induced with 300 μm isopropyl-β-D-thiogalactoside (IPTG) was incubated overnight. Upon centrifugation, the bacterial pellet was resuspended in HEPES buffer (20 mM HEPES, 150 mM KCl, 3% glycerol, pH 7.5). The pellet was lysed with ultrasonication, and the protein was purified with Ni^2+^ columns (R901-01) from Invitrogen (Waltham, MA, USA). Non-specific proteins were removed by washing the column with 20 mM imidazole in HEPES buffer. Protein was eluted from the column with 300 mM imidazole in HEPES buffer. After affinity purification, the protein was further purified with gel filtration chromatography. The concentrated protein was loaded onto a Superdex 200 column obtained from GE Healthcare (Chicago, IL, USA) coupled to an Akta FPLC purifier obtained from Cytiva Life Science (Marlborough, MA, USA). The peak fractions were collected with manual fraction collection and analyzed with SDS-PAGE (Figure S1). Fractions were aliquoted and flash-frozen in liquid nitrogen and stored in a cryo-freezer (−80 °C).

### 2.3. Enzyme Solution Preparation

A 5% (*w*/*v*) hemicellulase enzyme solution containing 0.2 M mannitol, 20 mM MES (pH 5.7), and 20 mM KCl was prepared. The solution was incubated in a water bath at 55 °C for 10 min to inactivate proteases and enhance enzyme solubility. The solution was then cooled to room temperature (25 °C) and 0.1% (*w*/*v*) BSA and 10 mM CaCl_2_ were added. The enzyme solution was prepared fresh. The biosynthesis of cell wall polysaccharides is fueled by carbon fixed by solar energy during photosynthesis. We hypothesized that photosynthesis could be inhibited upon switching from daylight to far-red light [17]. Reduced photosynthesis may lower carbon allocation for cell wall biosynthesis, potentially allowing proteins to diffuse into deeper cell layers.

### 2.4. Plant Seedling Preparation

*Arabidopsis thaliana* (Col-0) seeds were sterilized with 70% ethanol for 15 min and then 100% ethanol for 5 min. The seeds were dried in a laminar flow hood for 30 min and sowed in a sterile plate containing autoclaved ¼ MS liquid medium without sugar. The absence of sugar in the growth medium was useful not only for inhibiting the growth of fungus but also for minimizing the carbon source that is required for the biosynthesis of cell wall polysaccharides. Seeds were exposed to daylight or far-red light for 48 h after overnight daylight exposure (12–15 h) at 25 °C.

### 2.5. Partial Enzymatic Digestion of Cell Wall and Peptide Treatment

Six seedlings for each condition were incubated in 5, 10, and 20% enzyme solutions for 4, 6, 12, and 24 h in dark and washed three times with rinsing buffer containing 0.2 M Mannitol, 4 mM MES (pH 5.7), and 15 mM MgCl_2_. The timing of 4 h enzyme pretreatment was determined based on a standard protocol for preparing Arabidopsis mesophyll protoplasts. The seedlings were dried on filter paper and submerged in protein solution (1 mg/mL in HEPES buffer). The seedlings were incubated in dark at 25 °C for 4, 8, and 12 h [18]. The seedlings were rinsed with DI water and then viewed using a Leica Confocal SP8-SMD microscope (Wetzlar, Germany) with a laser excitation wavelength of 488 nm.

### 2.6. Confocal Imaging

Confocal imaging was performed with a Leica TSC SP8 point scanning confocal microscope equipped with a white light laser (WLL) exciting at 488 nm. The emission for every pixel was detected with a single-channel PMT detector. The range was set between 498 nm to 530 nm using an acousto-optical tunable filter (AOTF). The image resolution was 0.24 μm in X and Y and 1 μm in Z. The scanning speed was set to 400 lines per second. Brightfield images were acquired by a different PMT detector measuring 488 nm transmitted light. All representative confocal images used in this study were selected from at least 6 treated seedlings for each test condition. As another piece of supporting evidence for NLS activity: while some cytosolic GFP localizations were observed, no GFP signals were found in the nucleus (Figure S2).

## 3. Results

**Figure 1:**
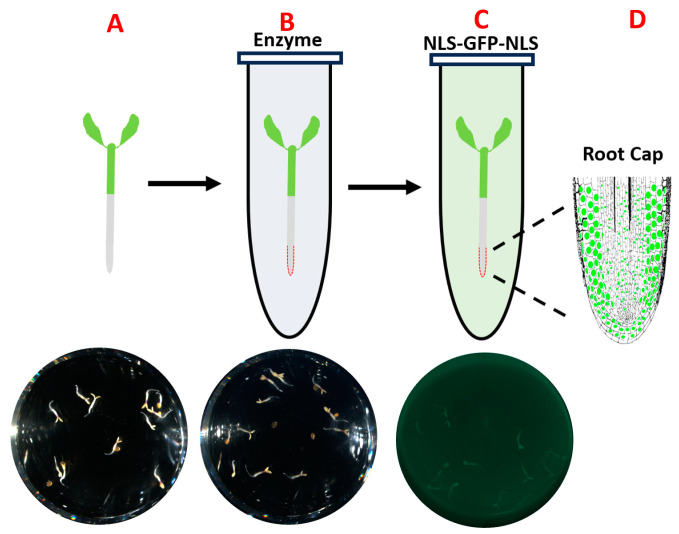
Schematic overview of nuclear protein delivery through the combination of partial cell wall digestion and nuclear localization sequence (NLS). (**A**) Wild-type Arabidopsis seedlings grown under daylight or far-red light. (**B**) The seedlings incubated in hemicellulase solution. (**C**) The seedlings incubated in protein solution. (**D**) Post-incubation, seedlings are imaged on a confocal laser scan microscope to confirm nuclear delivery.

In this technical report, we present a new approach to achieve the goal of protein delivery in walled plant cells, as evidenced by GFP signal in the nucleus that is greater than the cytoplasmic signal. This is accomplished by partially digesting the cell wall of young *Arabidopsis thaliana* (Col-0) seedlings with enzyme (PECWD, partial enzymatic cell wall digestion), allowing cargo proteins to enter the cytosol of plant cells (Figure 1A–C), in a way that is analogous to the protoplast transformation [19]. Here we show that the addition of two flanking NLS motifs can help to localize the GFP or GFP-tagged cargos to the nucleus, producing a round and uniform object that is amenable to image analysis and provides unambiguous confirmation and comparison of successful intracellular protein delivery (Figure 1D) [19,20].

**Figure 2:**
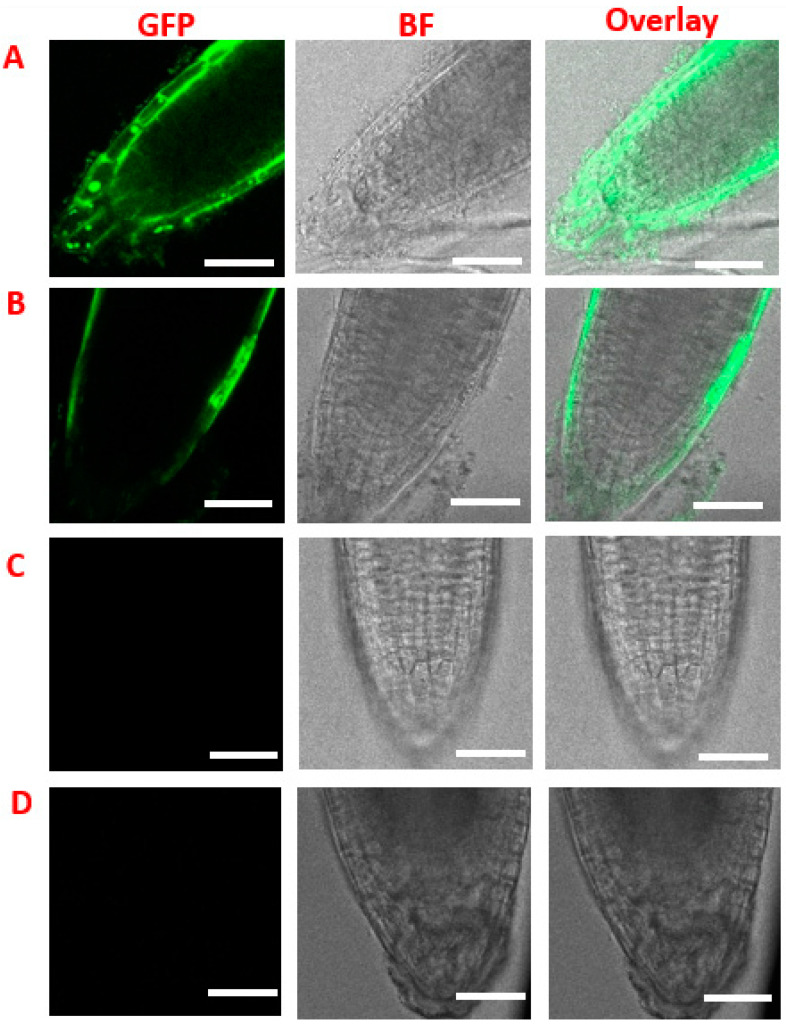
Nuclear internalization of GFP peptide in light-grown Arabidopsis seedlings. (**A**) Hemicellulase-digested seedlings incubated with 1 mg/mL NLS-GFP-NLS-His for 12 h. (**B**) Intact seedlings incubated with 1 mg/mL NLS-GFP-NLS-His. (**C**) Hemicellulase-digested seedlings without peptide incubation. (**D**) Intact seedlings without enzyme digestion and peptide incubation. The scale bars are 40 µm.

Intense nuclear GFP fluorescence signals (round green nucleus) were detected from the epidermal cell layers of cone-shaped root cap of seedlings pretreated with hemicellulase and incubated with NLS-GFP-NLS-His (Figure 2A), confirming the integrity and functionality of the imported GFP proteins in the nucleus. Without enzyme pretreatment (Figure 2B) or protein incubation (Figure 2C), no nuclear GFP fluorescence signal was observed. Moreover, shorter protein incubation times of 4 h and 8 h did not yield any detectable nuclear GFP signals (Figure S3). Importantly, the seedlings remained intact post-enzyme and peptide treatment, as evidenced by the zoom-out images capturing the entire root (Figures S4 and S5). These results, together with the intense green fluorescence exhibited by Fluorescein diacetate (FDA)-treated plant cells (Figure S6), confirm that the viability of seedlings is not affected by our treatment. It is worth noting that no positive nuclear GFP localization was detected in other tissues including the root elongation zone, root trichome, root hair, hypocotyl, and cotyledon (Figure S7), presumably due to defective cell wall biosynthesis on fast-dividing root cap cells [21]. In addition to hemicellulose, we also explored the potential of other cell wall-degrading enzymes that can serve as candidates for PECWD. While the use of pectinase and macerozyme results in the removal of the root cap (Figure S8), no nuclear GFP fluorescence was detected on seedlings pretreated with cutinase (Figure S9). It should be noted that cutinase enzyme breaks down the cuticle, which serves as a protective and hydrophobic barrier of the external surface of plant tissues. Cuticles in glandular trichomes contain discontinuities such as cuticular gaps, cuticular pores, and cuticular holes [22,23,24,25,26]. Together these results support our hypothesis that partially digested crude cell walls have enhanced permeability to cargo proteins.

**Figure 3:**
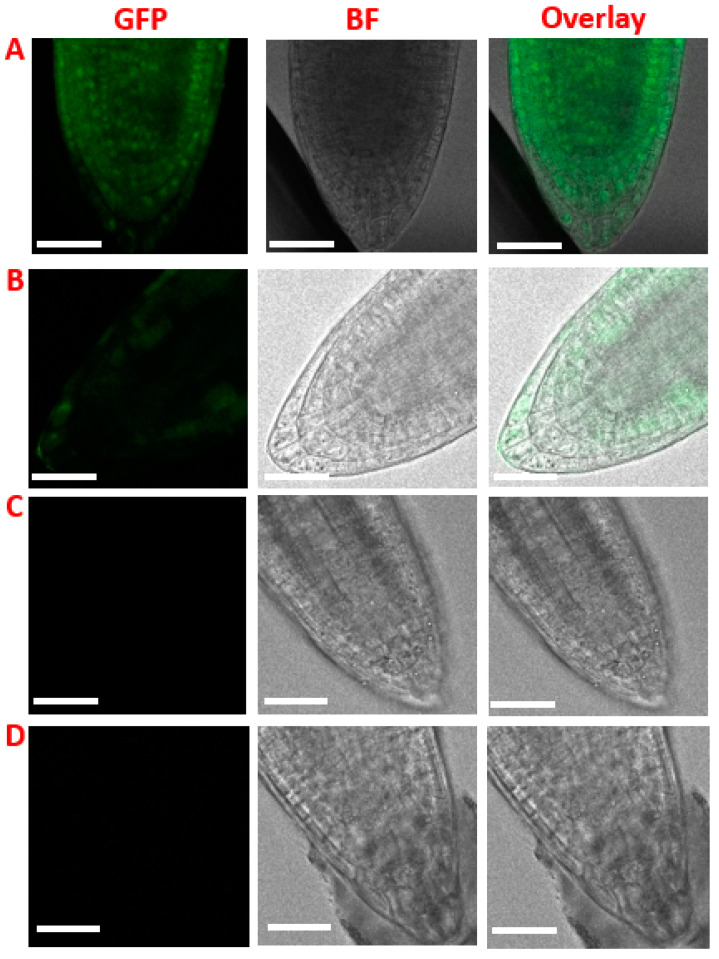
Nuclear internalization of GFP peptide in far-red grown Arabidopsis seedlings. (**A**) Hemicellulase-digested seedlings incubated with 1 mg/mL NLS-GFP-NLS-His for 12 h. (**B**) Intact seedlings incubated with 1 mg/mL NLS-GFP-NLS-His. (**C**) Hemicellulase-digested seedlings without peptide incubation. (**D**) Intact seedlings without enzyme digestion and peptide incubation. The scale bars are 40 µm.

After validating PECWD for quantitative protein delivery on epidermal cell layers, we next assessed whether our approach could be further exploited to deliver the protein into deeper cell layers where meristematic cells are embedded. After incubating hemicellulose-digested far-red grown Arabidopsis seedlings with 1 mg/mL NLS-GFP-NLS-His solution for 12 h, both confocal (Figure 3A) and 3D images reconstructed by the Z-stack images (Figure S10) revealed that nuclear GFP fluorescence signals were detected throughout all cell layers of the cone-shaped root cap. Similar to the light-grown Arabidopsis seedling, far-red grown seedlings, when not pretreated with hemicellulase or incubated with protein solution, showed no nuclear GFP signals (Figure 3B,C). It should be noted that longer growth time (6 days and 9 days) did not result in the nuclear delivery of GFP proteins (Figure S11). These results align with previous findings that far-red light exposure can alter the morphological and physiological properties of Arabidopsis root [17,27]. PECWD, when coupled with biological modifications of plant tissues, may offer a new approach to engineer meristematic cells and generate stably transformed plants in a culture-free manner [20].

**Figure 4:**
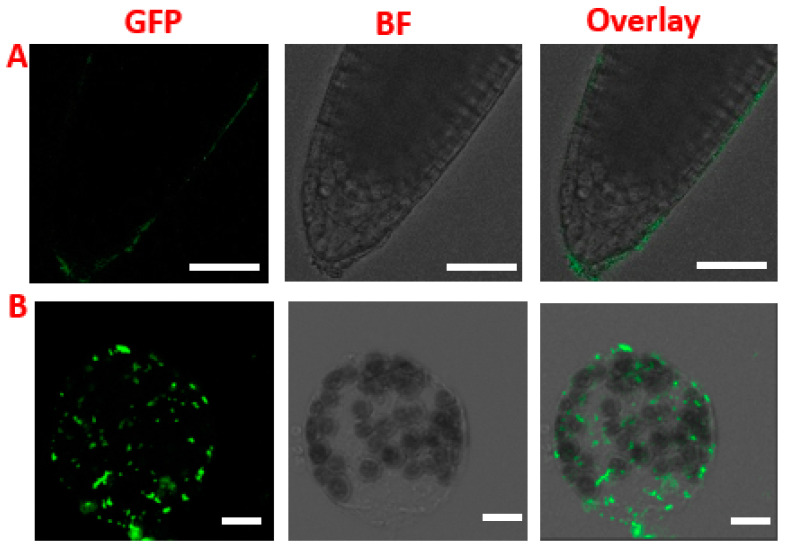
Nuclear internalization of GFP-tagged Cas9 peptide in far-red grown Arabidopsis seedlings. (**A**) Hemicellulase-digested seedlings incubated with 1 mg/mL GFP-tagged Cas9 solution. The scale bars are 40 µm. (**B**) Arabidopsis protoplast incubated with 1 mg/mL GFP-tagged Cas9 solution. The scale bars are 10 µm.

While the above strategy is useful, the delivery of larger proteins is required to facilitate the major goals of plant delivery, such as gene editing. This motivated our design of a Cas9 version of recombinant protein containing a GFP-tagged Cas9 flanked by two NLS, with a molecular weight of 188 kDa almost seven times as large as GFP. As indicated in Figure 4A, incubation with GFP-tagged Cas9 solution results in no GFP localization, whereas intensive nuclear GFP fluorescence signals were detected in Arabidopsis protoplasts with fully digested cell wall prepared by a standard procedure (Figure 4B) [2,19]. It should be noted that longer digestion times and higher enzyme concentrations did not result in the delivery of Cas9 protein into the plant cell (Figure S12).

## 4. Discussion

Our study provides the first piece of evidence that partial digestion of the plant cell wall enables direct protein delivery into plant cells. The two flanking NLS sequences were used to improve the nuclear delivery of GFP proteins, enabling unambiguous confirmation and comparison of successful intracellular protein delivery. However, our findings are a departure from the prevailing assumption that efficient delivery of proteins only occurs when the cell wall is completely removed. Two factors could be responsible for the discrepancy. As existing plant transformation methods mostly rely on protoplast culture (PEG, liposome, and electroporation) or physical disruption of the cell wall (particle bombardment, microinjection, and needle puncture), methods involving an “intermediate state” at which the cell wall is partially degraded have yet to be explored. Our PECWD represents an ideal way to explore the degree to which this “intermediate state” can be tuned to improve protein delivery efficiency. In addition, the majority of emerging cell-penetrating peptide (CPP)-based [13] and nanoparticle (NP)-based [15,28] techniques often suffer from low reproducibility. While the exact origins of these variabilities remain unclear, they are fundamentally connected to the structure of the plant cell wall and the resulting complex interactions that influence intercellular and intracellular protein translocation. The use of enzymatic pretreatment may help to overcome the obstacle of low efficiency and reproducibility associated with CPP- or NP-mediated uptake of proteins, potentially expanding the utility of nanotechnology in many applications to meet the increasing demand for the sustainable production of foods, materials, and energy. It should be noted that higher enzyme concentration and longer digestion period did not result in larger protein (Cas9) localization in digested seedlings. Such discrepancy can be readily explained by the incomplete cell wall removal during the PECWD process, which rejects large Cas9 proteins while allowing small GFP proteins to pass through [10,11,13,14]. As the aim of PECWD is to improve the permeability without losing the overall integrity and stability of the cell wall structure, a size exclusion limit should be expected. Future studies that utilize lipid- and polymer-based nanoparticles to encapsulate and condense proteins can serve to address this limitation [11,29,30]. This novel approach may have broad utilities in plant transformation techniques that require the introduction of macromolecules and may enable tissue culture-free gene editing methods.

The concept of utilizing a PECWD may be combined with various types of nanomaterials [11,29,30], expanding the utility of nanotechnology beyond the plant cell wall barrier and the current stage of proof-of-concept [15,29,30]. In addition to a model dicot plant Arabidopsis, our approach of partially disrupting cell wall polysaccharides can potentially transform the field of plant biology and plant biotechnology by creating versatile and efficient transformation methods applicable to a wide range of plant species. Besides the use of PECWD in nanotechnology, the combination of PECWD with the existing plant biotechnologies, such as bombardment-mediated transformation, may be a future direction. The composition and structure of plant cell walls can differ among species, cell types, and development stages, necessitating optimizations of enzyme type, enzyme concentration, and digestion time on a case-by-case manner. To ensure uniform treatment across different tissues, the enzyme digestion must be performed under controlled and isolated physical conditions including pH, temperature, pressure, humidity and illumination. Although our method is limited by the size of cargo proteins, the framework established here provides a crucial starting point for more complex applications. For example, future studies that increase the cargo size of our delivery system may allow direct delivery of sgRNA-Cas9 complex or plasmids into the germ cells in the shoot meristem, resulting in stably transformed plants. In addition, methods can be developed to target the plant meristem via enzyme digestion, facilitating easier genomic transformations through partially digesting the plant meristem and thus increasing the efficiency of protein delivery. It is also essential to systematically investigate the compatibility of cargo proteins with a range of target plant species, as well as different types of tissues, in order to expand the scope of our method. Ultimately, a molecular-level understanding of the structure–function relationship between the cell wall and the permeability will help to unravel the origin of variabilities observed in many biomolecule delivery methods [31,32,33], paving the way toward rational engineering of plants.

## Data Availability

Data are available upon request.

## Supplementary Materials

The following supporting information can be downloaded at: https://www.mdpi.com/article/10.3390/cimb46120870/s1, Figure S1. Protein purification steps and SDS-PAGE of purified recombinant proteins. Figure S2. Hemicellulase digested daylight grown Arabidopsis seedlings incubated with 1 mg/mL GFP-His solution. Figure S3. Nuclear internalization of GFP peptide in far-red grown Arabidopsis seedlings at different incubation times. Figure S4. Nuclear internalization of GFP peptide in light-grown Arabidopsis seedlings. Figure S5. Nuclear internalization of GFP peptide in far-red grown Arabidopsis seedlings. Figure S6. Hemicellulase digested Arabidopsis seedlings stained with Fluorescein Diacetate (FDA). Figure S7. Hemicellulase digested Arabidopsis seedlings incubated with 1 mg/mL NLS-GFP-NLS-His solution. Figure S8. Far-red grown Arabidopsis seedlings incubated with Pectinase, Macerozyme and Hemicellulase at different enzyme concentrations. Figure S9. Cutinase digested Arabidopsis seedlings incubated with 1 mg/mL NLS-GFP-NLS-His solution. Figure S10. Nuclear internalization of GFP peptide in far-red grown Arabidopsis seedlings. Figure S11. Nuclear internalization of GFP peptide in far-red grown Arabidopsis seedlings. Figure S12. Hemicellulase digested Arabidopsis seedlings incubated with 1 mg/mL NLS-Cas9-NLS-GFP-Motif-His solution.
