# Supplementary material for: Direct Nuclear Delivery of Proteins on Living Plant via Partial Enzymatic Cell Wall Digestion
Source: Curr Issues Mol Biol. 2024 Dec 23;46(12):14487–96. doi: 10.3390/cimb46120870 (PMC11674260; doi:10.3390/cimb46120870)
Supplement: Supplementary file 1 [file cimb-46-00870-s001.zip › cimb-3364046-supplementary.pdf]

Supplementary Information for

Direct Nuclear Delivery of Proteins on Living Plant via Partial Enzymatic Cell Wall Digestion

Qufei Gu,[1,2,*,†] Nathan Ming,[1,†], Yalikunjiang Aizezi,[1,3] Xiaoyang Wei,[1] Yizhong Yuan,[1] Brian Esquivel[1] and Zhi-yong Wang[1,*]

[1]Department of Plant Biology, Carnegie Institution for Science, Stanford, California, 94305, USA

[2]Department of Physics, Stanford University, Stanford, CA 94305, USA

[3]Department of Biology, Stanford University, Stanford, CA 94305, USA

*** Email: zwang@carnegiescience.edu**, **qgu@carnegiescience.edu**

This PDF file includes:

Figures S1 to S12

	Protein Purification Steps
1	Resuspend the pellet in HEPES buffer
2	Lyse the pellet with ultrasonication
3	Flow the clarified lysate through the Ni^{2+} column
4	Washing with 20 mM imidazole in HEPES buffer
5	Elute with 300 mM imidazole in HEPES buffer
6	Gel filtration chromatography with manual fraction collection

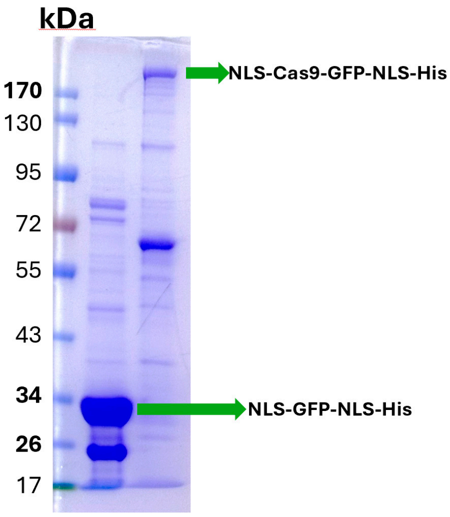

Figure S1. Protein purification steps and SDS-PAGE of purified recombinant proteins.

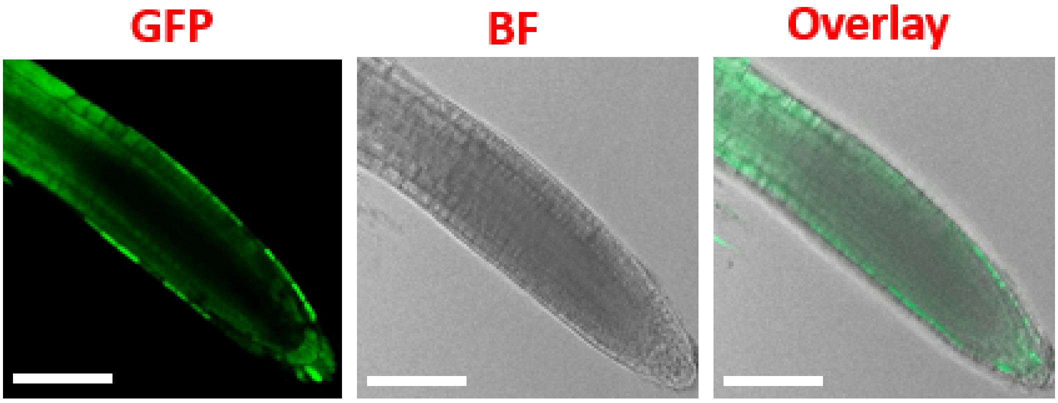

Figure S2. **Hemicellulase digested daylight grown Arabidopsis seedlings incubated with 1 mg/mL GFP-His solution.** The scale bars in insets are 50 µm.

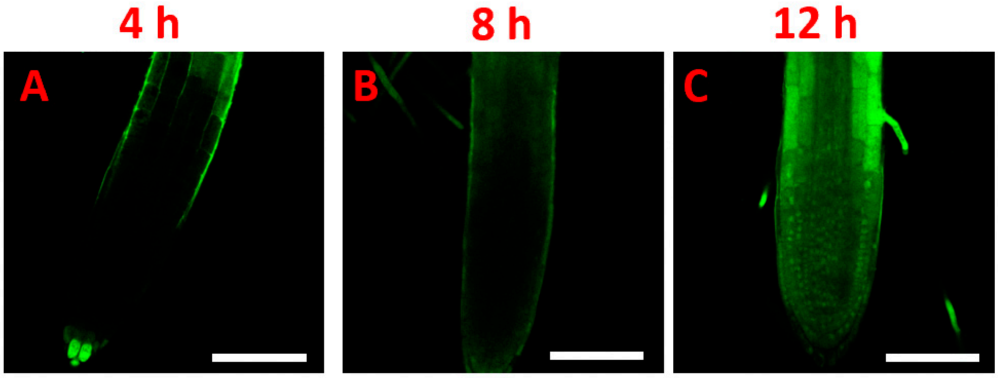

Figure S3. Nuclear internalization of GFP peptide in far-red grown Arabidopsis seedlings at different incubation times. (A) Hemicellulase digested seedlings incubated with 1 mg/mL NLS-GFP-NLS-His for 4 h. (B) Hemicellulase digested seedlings incubated with 1 mg/mL NLS-GFP-NLS-His for 8 h. (C) Hemicellulase digested seedlings incubated with 1 mg/mL NLS-GFP-NLS-His for 12 h. The scale bars are 50 µm.

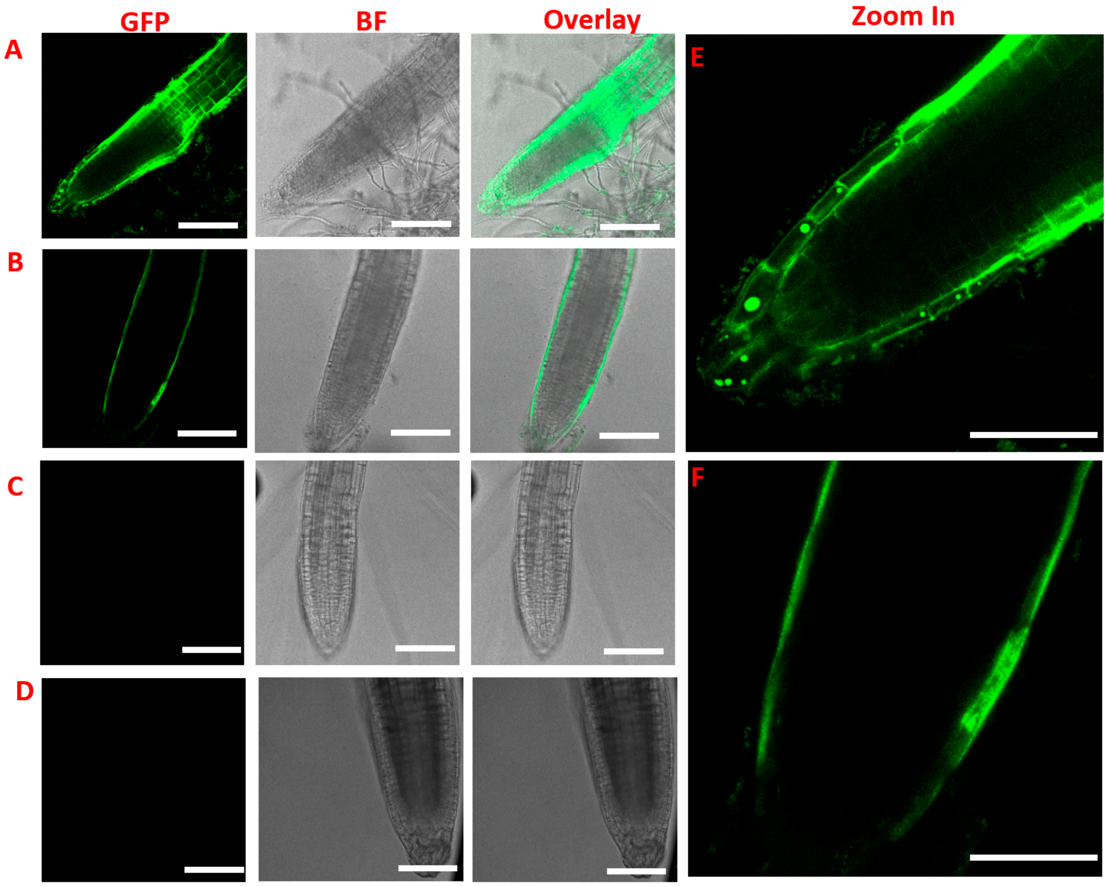

Figure S4. Nuclear internalization of GFP peptide in light-grown Arabidopsis seedlings. (A) Hemicellulase digested seedlings incubated with 1 mg/mL NLS-GFP-NLS-His for 12 h. (B) Intact seedlings incubated with 1 mg/mL NLS-GFP-NLS-His. (C) Hemicellulase digested seedlings without peptide incubation. (D) Intact seedlings without enzyme digestion and peptide incubation. The scale bars are 50 µm. (E) Zoom-in from panel A showing nuclear GFP signals in root cap as the result of successful delivery. (F) Zoom-in from panel B showing cytosolic GFP signals only. The scale bars are 100 µm.

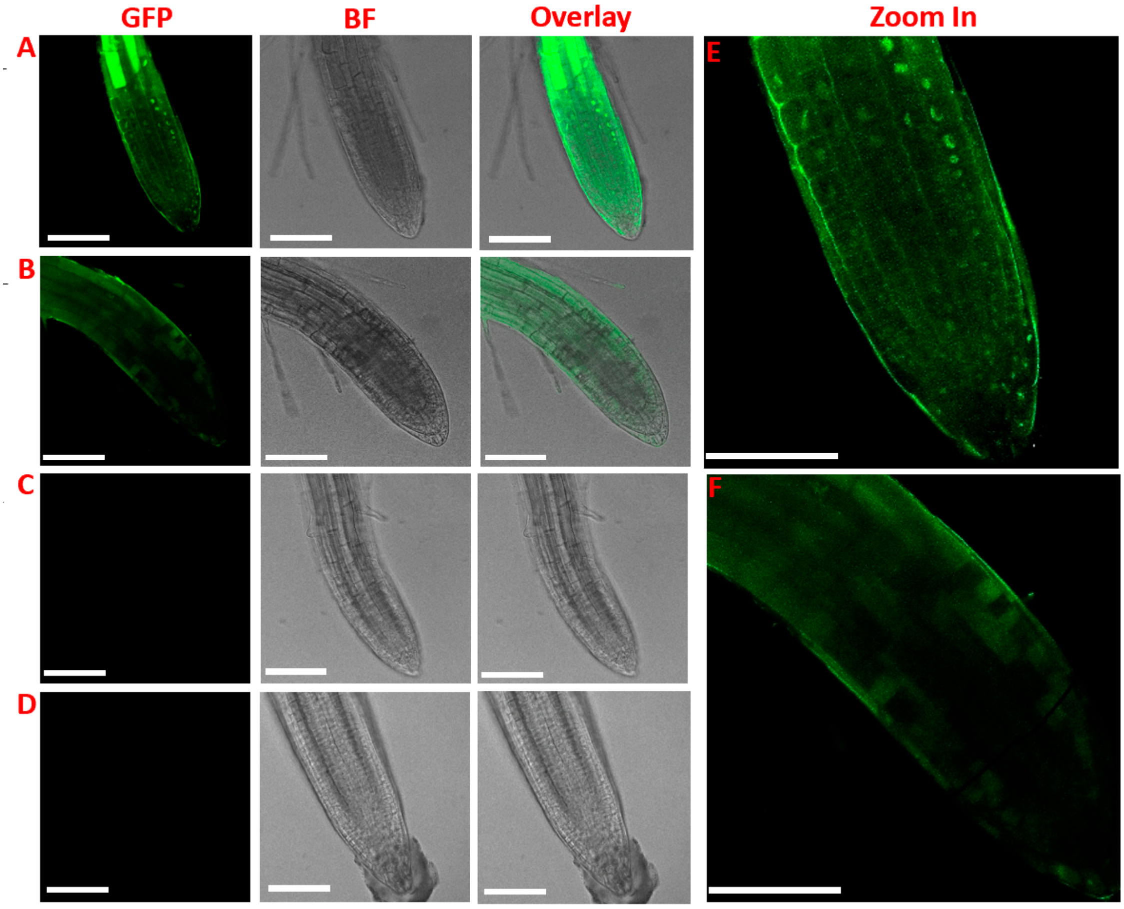

Figure S5. Nuclear internalization of GFP peptide in far-red grown Arabidopsis seedlings. (A) Hemicellulase digested seedlings incubated with 1 mg/mL NLS-GFP-NLS-His for 12 h. (B) Intact seedlings incubated with 1 mg/mL NLS-GFP-NLS-His. (C) Hemicellulase digested seedlings without peptide incubation. (D) Intact seedlings without enzyme digestion and peptide incubation. The scale bars are 50 µm. (E) Zoom-in from panel A showing nuclear GFP signals in the root cap as the result of successful delivery. (F) Zoom-in from panel B showing cytosolic GFP signals only. The scale bars are 100 µm.

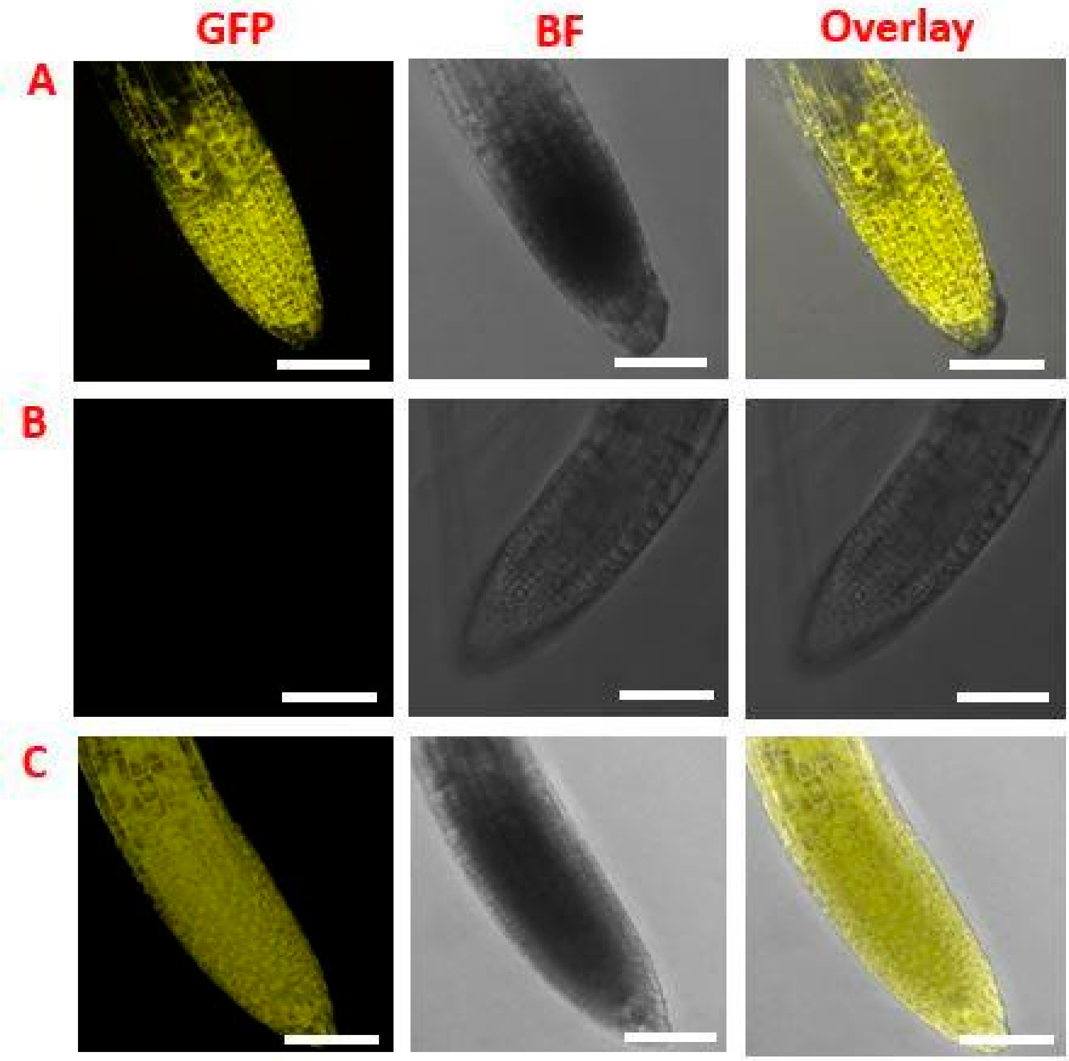

Figure S6. Hemicellulase digested Arabidopsis seedlings stained with Fluorescein Diacetate (FDA). (A) Hemicellulase digested seedlings stained with FDA for 15 min. (B) Hemicellulase digested seedlings without FDA staining. (C) Untreated seedlings stained with FDA for 15 min. The scale bars are 50 µm.

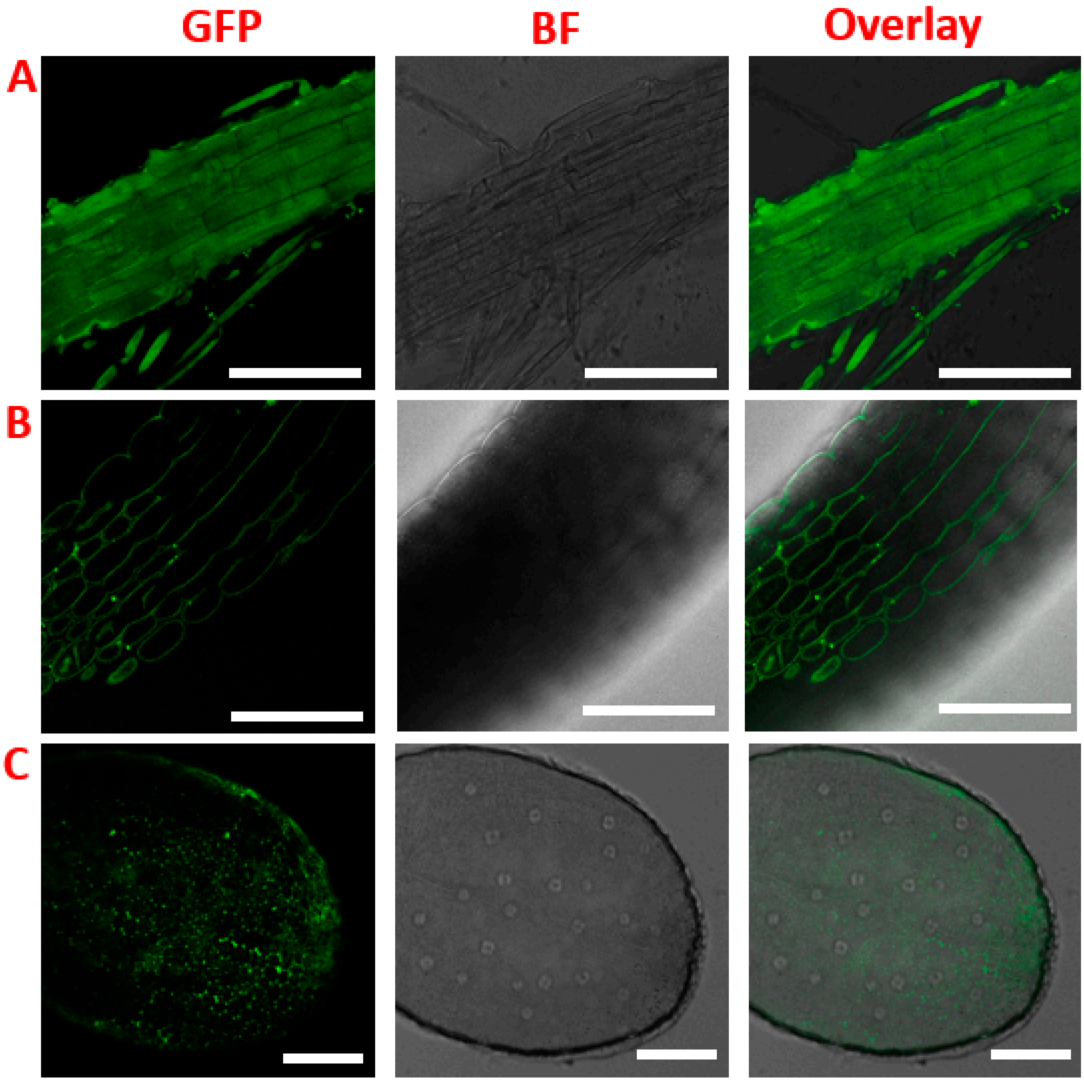

Figure S7. Hemicellulase digested Arabidopsis seedlings incubated with 1 mg/mL NLS-GFP-NLS-His solution. Representative confocal images of (A) root elongation zone, root hair, root trichome, (B) hypocotyl and (C) cotyledon. The scale bars are 100 µm.

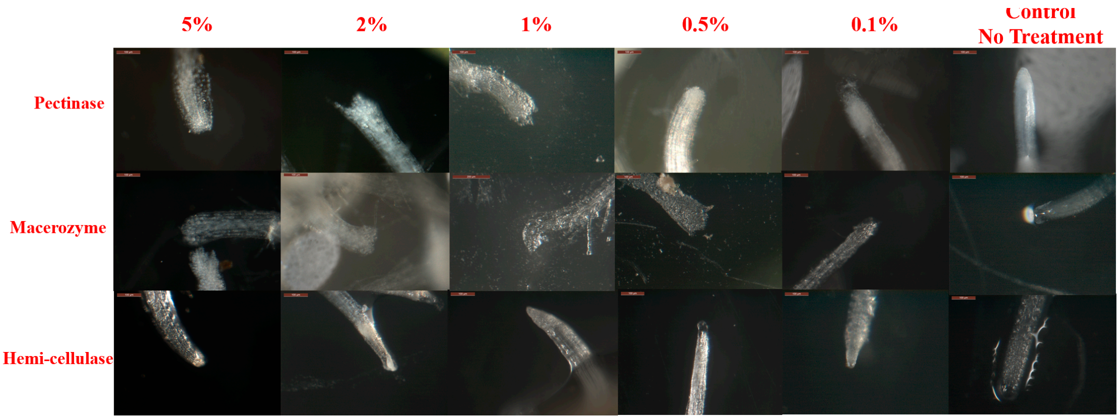

Figure S8. Far-red grown Arabidopsis seedlings incubated with (A) Pectinase, (B) Macerozyme and (C) Hemicellulase at different enzyme concentrations.

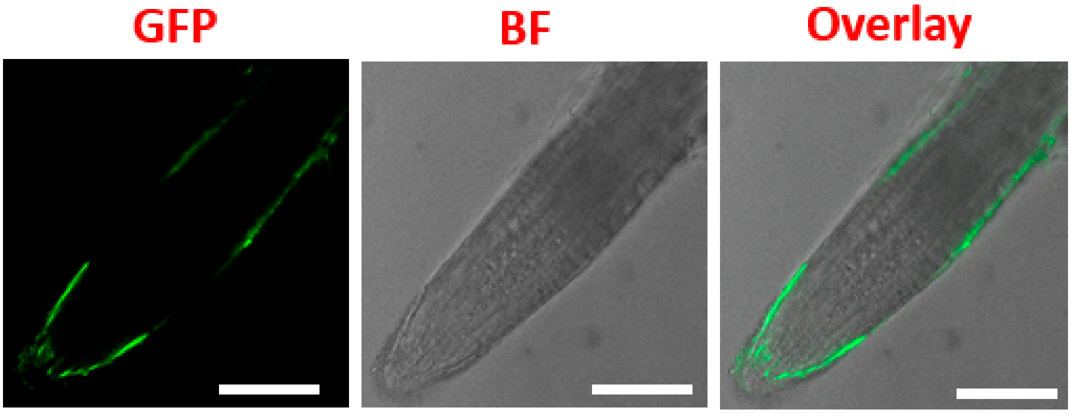

Figure S9. Cutinase digested Arabidopsis seedlings incubated with 1 mg/mL NLS-GFP-NLS-His solution. The scale bars are 50 µm.

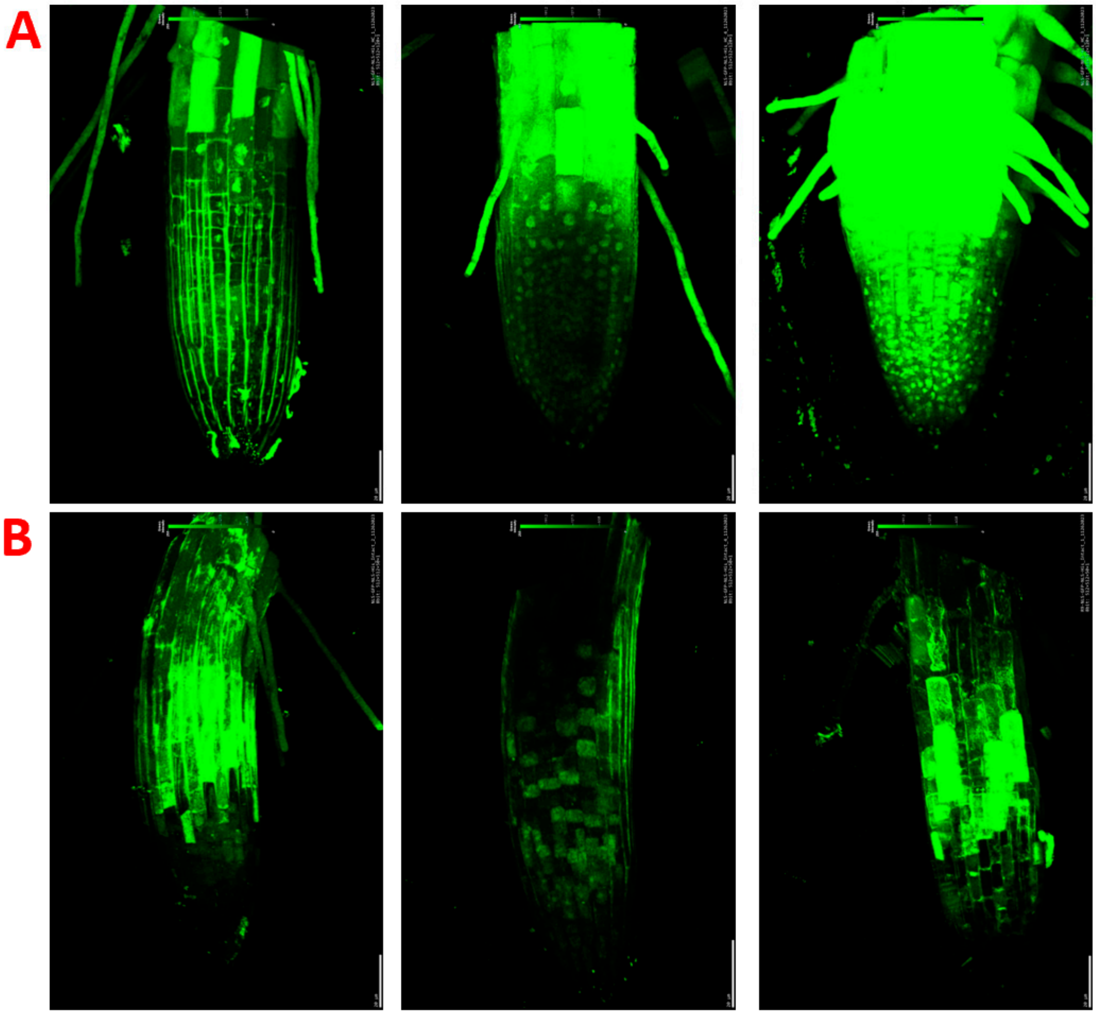

Figure S10. Nuclear internalization of GFP peptide in far-red grown Arabidopsis seedlings. 3D images reconstructed by the Z-stack images (A) Hemicellulase digested seedlings incubated with 1 mg/mL NLS-GFP-NLS-His for 12 h**.** (B) Intact seedlings incubated with 1 mg/mL NLS-GFP-NLS-His for 12 h.

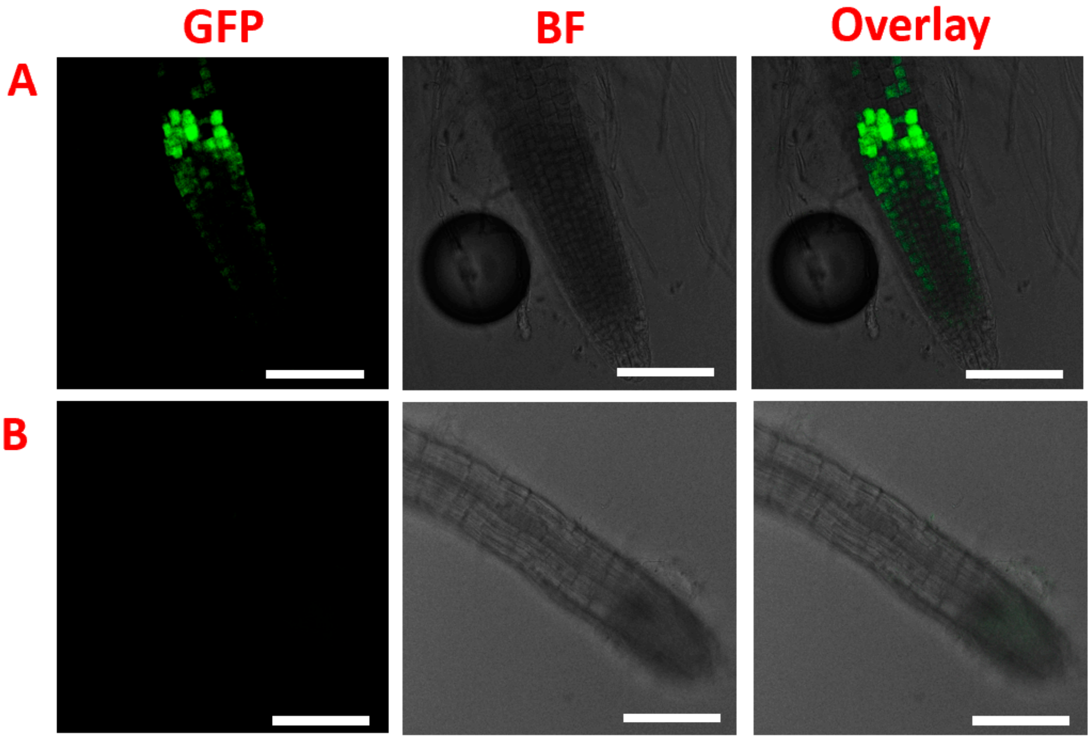

Figure S11. Nuclear internalization of GFP peptide in far-red grown Arabidopsis seedlings. (A) Hemicellulase digested seedlings (6-day-old) incubated with 1 mg/mL NLS-GFP-NLS-His for 12 h. (B) (A) Hemicellulase digested seedlings (9-day-old) incubated with 1 mg/mL NLS-GFP-NLS-His for 12 h. The scale bars are 40 µm.

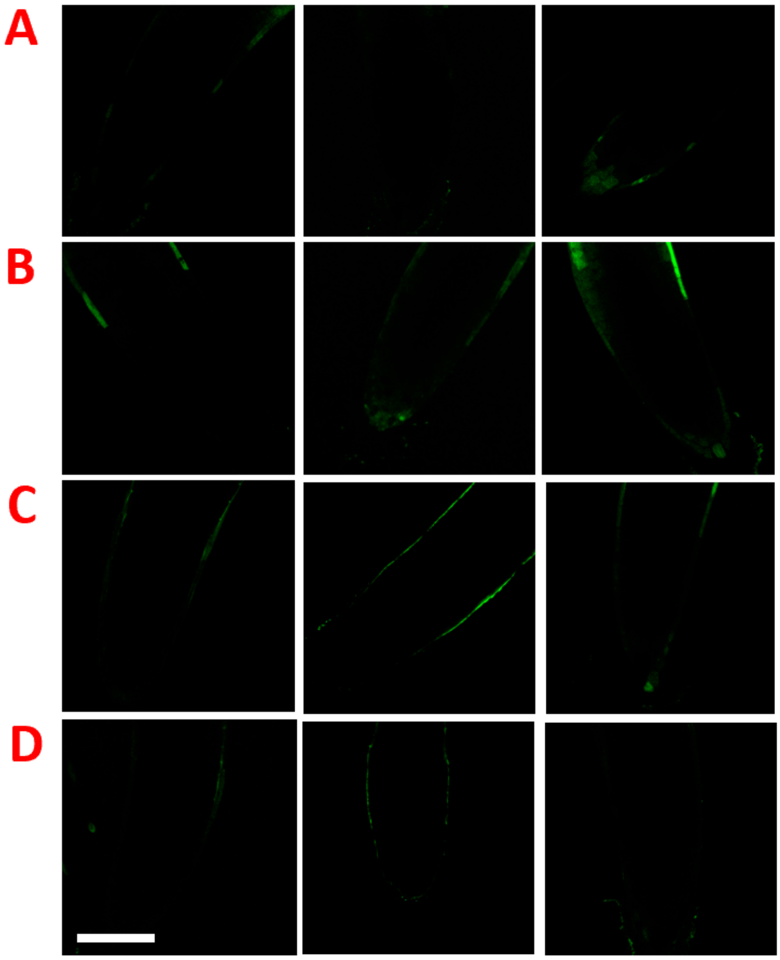

Figure S12. Hemicellulase digested Arabidopsis seedlings incubated with 1 mg/mL NLS-Cas9-NLS-GFP-Motif-His solution. Representative confocal images of far-red grown Arabidopsis seedlings digested by (A) 0%, (B) 5%, (C) 10%, (D) 20% Hemicellulase for 6 h (left panel), 12 h (middle panel) and 24 h (right panel).
